# Clinical Annotation and Segmentation Tool (CAST) Implementation for Dental Diagnostics

**DOI:** 10.7759/cureus.48734

**Published:** 2023-11-13

**Authors:** Taseef H Farook, Farhan H Saad, Saif Ahmed, James Dudley

**Affiliations:** 1 Adelaide Dental School, University of Adelaide, Adelaide, AUS; 2 Department of Electrical and Computer Engineering, North South University, Dhaka, BGD

**Keywords:** deep learning, clinical photography, radiology, digital dentistry, artificial intelligence

## Abstract

Purpose

This study aims to document the early stages of development of an unsupervised, deep learning-based clinical annotation and segmentation tool (CAST) capable of isolating clinically significant teeth in both intraoral photographs and their corresponding oral radiographs.

Methods

The dataset consisted of 172 intraoral photographs and 424 dental radiographs, manually annotated by two operators, augmented to yield 6258 images for training, 183 for validation, and 98 for testing. The training involved the use of an object detection model (‘YOLOv8’) combined with a feature extraction system (‘Segment Anything Model’). This combination enabled the auto-annotation and segmentation of tooth-related features and lesions in both types of images without operator intervention. Outputs were further processed using a data relabelling tool (‘X-AnyLabeling’) enabling the option to manually reannotate erroneous data outputs through reinforcement learning.

Results

The trained object detection model achieved a mean average precision (mAP) of 77.4%, with precision and recall rates of 75.0% and 72.1%, respectively. The model was able to segment features from oral images annotated by polygonal boundaries better than radiological images annotated using bounding boxes.

Conclusion

The development of the auto-annotation and segmentation tool showed initial promise in automating the image labelling and segmentation process for intraoral images and radiographs. Further work is required to address the limitations.

## Introduction

When integrating computer vision-based artificial intelligence (AI) into the realm of medical and dental imaging, the process begins with image preprocessing to reduce noise and artifacts, followed by training AI models through the labelling of initial datasets by human operators [1]. This labelling process, referred to as annotation, is a supervised task predominantly executed by human clinicians and operators. However, a significant challenge in the implementation of AI in medical datasets stems from the necessity of operator-dependent image labelling, influenced by their clinical experience, thus introducing potential biases [2,3].

A recurring issue is the varying classification methods used to label datasets over time. Until recently, most datasets were labelled using bounding boxes while recent research adopts the use of robust polygonal boundaries. Each labelling approach offers its own set of advantages and limitations. For context, we have provided, in the Materials and Methods section, a visual representation of the challenges encountered when annotating and segmenting using bounding boxes and the discrepancies that emerge across operators when classifying dental datasets. Annotation refers to marking the regions of interest where bounding boxes are easily placed at the expense of accurate margin recreation while polygon boundaries can recreate the specific margins of the tooth, but the marking is heavily dependent on the operators’ best judgement of where the margins lie. Furthermore, revisiting the annotations of older datasets often proves challenging due to the reliance on human operators and their specific methods applied for the segmentation purpose. The task is further complicated if the reannotation has to be carried out by the same operators who initially labelled the datasets. Inconsistent availability of codebooks, challenges in reconciling changes to coding schemes over time, uncertainties about data source reliability, and poorly documented alterations in data collection and storage procedures pose obstacles in data analysis [4].

As new computer vision architectures, models, and software are consistently released, some methods become unsupported in previous implementations and dataset annotation formats become redundant [5]. Correction in several instances requires hours of human interventions by clinical operators. Another challenge lies in the classification and annotation of clinical features, such as dental caries (decayed teeth), across diverse datasets using a single model or workflow. Often, multiple models and workflows become necessary to label datasets, leading to compatibility issues as newer iterations emerge. Consequently, two distinct problems warrant attention: improving data annotation to identify clinical areas of interest and developing effective segmentation methods that operate autonomously, mitigating the aforementioned issues.

A potential resolution for both these challenges involves introducing an unsupervised clinical auto-annotation and segmentation tool (CAST) that eliminates the need for human operators to manually annotate images and can consistently reannotate datasets if required for newer workflows. Unsupervised learning in the current context refers to the ability of an AI algorithm to read and process clinical images not previously labelled by clinical practitioners for regions of interest, as opposed to supervised learning where the AI model is only capable of identifying structures previously labelled on the dataset [6]. The ability of supervised learning is generally limited to specific types of data, such as radiographs and clinical images, whereas it is possible to amalgamate different datasets using a consolidated unsupervised AI. The approach of segmenting regions of interest without operator intervention entails teaching an unsupervised segmentation model using datasets labelled by human operators in the past and allowing it to refer to the said knowledge base when tasked with autonomously annotating and segmenting new unseen images. However, this strategy necessitates the use of robust object detection methods, a form of deep learning capable of learning from images, to initially train CAST. You Only Look Once (YOLO) has been a robust object detection method extensively used in previous research for the same task [7,8]. YOLOv8, the most recent iteration of YOLO introduced in 2023, surpassed its predecessors, including YOLOv3, scaled YOLOv5, YOLOv6, and YOLOv7, in terms of precision and recall [9]. The application of YOLOv8 for object detection of dental images is innovative in itself, but the incorporation of various AI models to automate the segmentation process that follows has remained unexplored due to the need for meticulous supervision in previous AI-based segmentation methods.

In contrast to conventional AI-based segmentation approaches, Meta's Segment Anything Model (SAM), introduced as a research technique in 2023, was demonstrated to excel in autonomously segmenting objects of diverse shapes, sizes, and instances within images, offering a more accurate comprehension of the objects present [10]. SAM has gained recognition in the computer vision research community for its potential to accurately segment objects within images using polygonal boundaries. This model employs semantic affinity to effectively manage partially obstructed and ambiguous scenarios, capitalising on a wide array of contextual visual information to achieve highly refined object segmentation [11]. In the current context, semantic affinity serves as an internal measure of how closely different pieces of visual information are related to one another.

However, no AI as of 2023 is perfect. To ensure a highly accurate clinical model while streamlining the workflow with minimal human intervention, there should be provisions for manual corrections in case of erroneous annotations or segmentations. For this purpose, the open-source software X-AnyLabeling, released in 2023, potentially offers the option to re-label with SAM. Such a modular tool can display generated annotations and can be programmed to allow human operators to rectify annotations, if necessary, thereby reinforcing accurate decisions [12]. To the authors’ best knowledge, this study represents the inaugural effort to amalgamate multiple deep learning models into an integrated automated workflow for annotating and segmenting dental datasets, with future provisions for manual error correction reinforcement. Therefore, the objective of this report was to document the early stages of development of an unsupervised deep learning-based annotation and segmentation model capable of isolating clinically significant teeth in both intraoral photographs and oral radiographs.

## Materials and methods

The study is a part of the Dental Loop initiative. The Dental Loop is a dedicated research-to-practice translatory initiative to shape AI into usable diagnostic aids in dental practice. Established in 2022, the initiative's objective is to create AI-based solutions for diverse clinical scenarios within the dental office. The Dental Loop aims to streamline these solutions into practical tools and workflows that clinicians can easily utilise while simultaneously offering patients AI-driven appointment scheduling and post-treatment follow-up support. The ultimate goal of the Dental Loop is to develop an AI dentist capable of empathising with and guiding patients at a level comparable to human dental practitioners. At a community level, the Dental Loop aims to alleviate the strain on public dental healthcare systems, reduce wait times to receive dental treatment, and provide convenient access to tailored private alternatives that suit the individual's unique circumstances [13]. To that extent, the Dental Loop received permission to conduct research and development using limited de-identified clinical and radiographic data from hospital dentistry. The research on the datasets was permitted by the University of Adelaide Human Research and Ethics Committee (HREC-2023-073) and North South University Institutional Review Board (2023/OR-NSU/IRB/0503).

The computational aspects of the current study were developed following the Python Enterprise Proposal-8 (PEP-8) guidelines. As the proposed workflow did not adhere to a conventional medical machine learning workflow, the study was reported according to the Checklist for Artificial Intelligence in Medical Imaging (CLAIM) guidelines [14] and reinforced following the Minimum Information for Clinical Artificial Intelligence Modelling (MI-CLAIM) protocol [15]. The study was designed to leverage the existing datasets that form the research foundation of the Dental Loop initiative. Its primary objective was to propose a workflow for the development of a clinical annotation and segmentation tool (CAST) built upon the Dental Loop framework.

**Figure 1:**
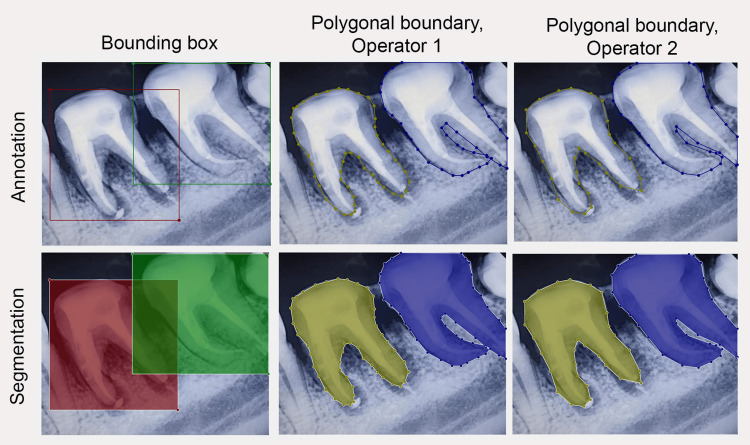
Visual comparison of manual annotation and segmentation using the bounding box and polygonal boundary approaches

Two distinct datasets were utilised for the present implementation. The first dataset encompassed 172 intraoral photographs of both upper and lower jaws. These images were meticulously annotated using polygonal classification methods by two operators to encompass 44 different classes, including Fédération Dentaire Internationale (FDI)-based tooth notations [16], carious and non-carious lesions, restorative status and the presence of fixed prostheses. The carious lesions were further categorized into ‘changes without visible cavitation,’ ‘changes with microcavitation,’ and ‘changes with cavitation,’ in accordance with established research on visual caries diagnostics [17]. Similarly, restorative status was subclassified into ‘amalgam restoration’ and ‘tooth-coloured restorations.’ The second dataset featured 424 digital peri-apical radiographs of teeth that had undergone complete or partial endodontic treatment. These images were categorized as ‘no endodontic treatment,’ ‘incomplete,’ ‘complete,’ or ‘endodontic mishap.’ It is worth noting that the datasets underwent classification using bounding boxes, balancing, and denoising procedures based on well-documented methodologies from a previous report [18]. Figure 1 demonstrates the different methods applied for labelling.

The data were subsequently divided into training, validation, and test sets with proportions of 70%, 20%, and 10%, respectively. Further data augmentation was applied, involving rotation within a range of -15 degrees to +15 degrees, alongside shearing effects, both vertically and horizontally at a 15-degree angle [19]. This augmentation process yielded a total of 6258 images for training, 183 images for validation, and 98 images for testing. In the training set, there were 890 images from the intraoral dataset, with the remaining 5368 sourced from the endodontic radiography dataset. In the test set, 25 images were from the intraoral dataset and 73 images were from the endodontic radiography dataset. Both of these datasets were processed using the YOLOv8 model.

The object detection and recognition phase incorporated the utilisation of the YOLOv8 model [20]. The process involved training the model and obtaining the weights for the intraoral dataset and radiography dataset, preparing the models for integration. This was followed by the Segment Anything Model (SAM), which constituted an essential component of the instance segmentation process. The model was constructed on the foundations of deep neural networks and specialized in the identification and delineation of individual objects within images. SAM's architecture encompassed various crucial components, namely the Backbone Network, Feature Pyramid Network, and Region Proposed Network. The segmentation process began with a Backbone Network, often using ResNet [21] or VGG [22], to extract fundamental features from images input into the system, forming the basis for subsequent segmentation. The Segmentation Head was responsible for the precise generation of instance masks, employing multiple layers and techniques. The Feature Pyramid Network (FPN) was then introduced to enhance object detection across multiple scales, capturing high-resolution details and semantic information to ensure accurate instance segmentation. Furthermore, the Region Proposal Network (RPN) played a role in generating region proposals, which identified potential object instances within images and guided the subsequent segmentation process to ensure precise delineation. SAM also incorporated an appropriate loss function, often combining instance segmentation losses like Mask R-CNN's Mask Loss with auxiliary losses to optimize the model during the training process.

**Figure 2:**
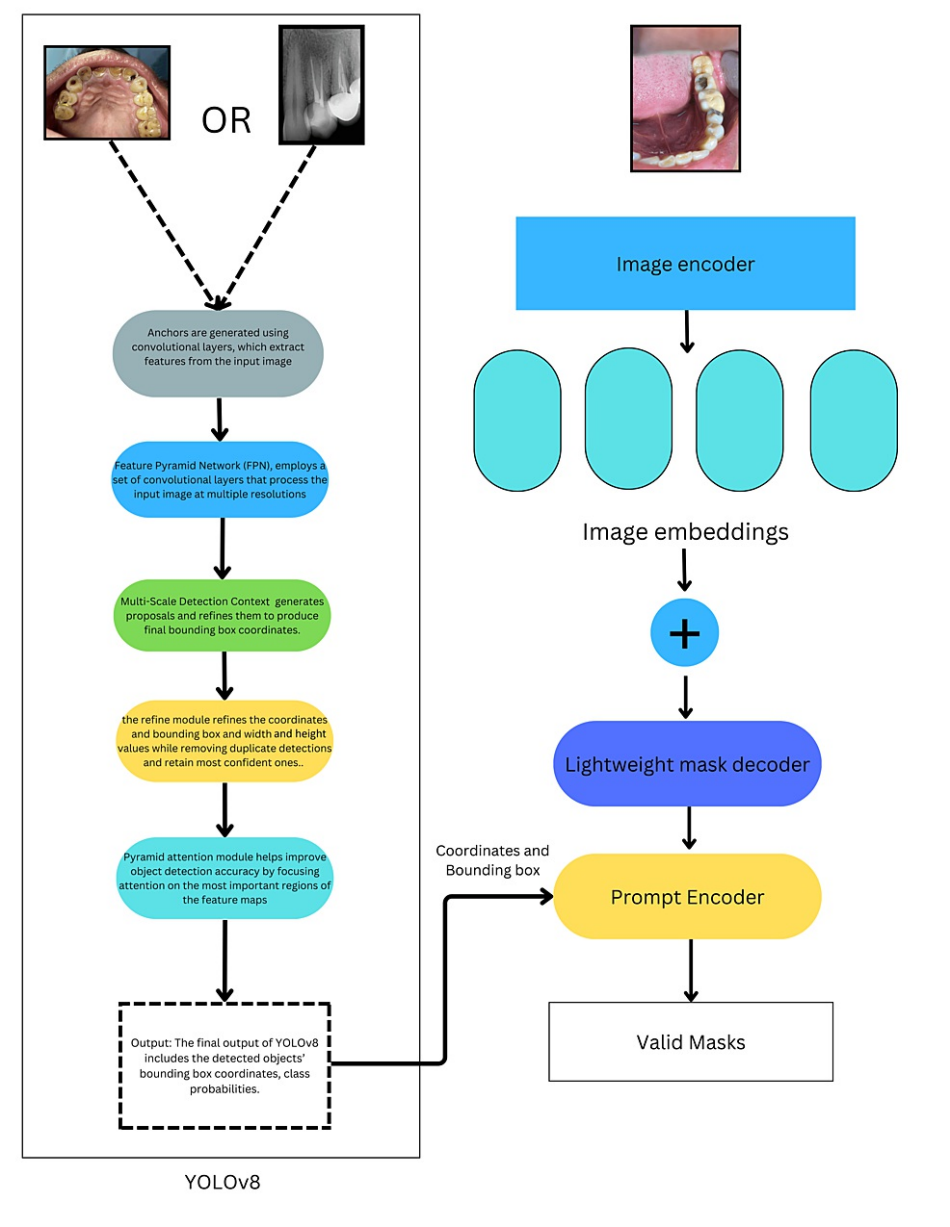
Workflow of the proposed clinical auto-annotation and segmentation tool

When encountering new, unseen data, the retrieved weights from the trained YOLOv8 model were employed in conjunction with the SAM model to streamline the labelling process. Following the application of SAM for automated instance segmentation and labelling, the initial annotations were in a .txt format. However, these annotations were subsequently converted into the more versatile and structured .json format, enhancing their accessibility and usability across various applications and tools. The utilization of X-AnyLabeling (https://github.com/CVHub520/X-AnyLabeling) facilitated the opening and display of all SAM Mask Generated Images. This allowed for a comprehensive view of the segmented objects, and the tool was the preferred choice for future research, enabling the reannotation of images through adjustments and movement of polygon points to ensure precise and accurate delineation of objects within the images. Figure 2 demonstrates the workflow in detail. To assess the system's performance, the mean average precision (mAP), precision, and recall were examined. Precision measures the ratio of correctly identified positive data (true positives) within the deep learning dataset compared to all correctly classified data. Recall, on the other hand, assesses the accuracy in identifying all positive data instances.

The mean average precision (mAP) is a widely employed metric for evaluating the object detection capabilities of computer vision models. This metric calculates a score by comparing the detected regions of interest to the ground truth. A higher mAP score indicates greater model accuracy. It is computed by determining the average precision (AP) for each class and then taking the average across all classes.

## Results

**Figure 3:**
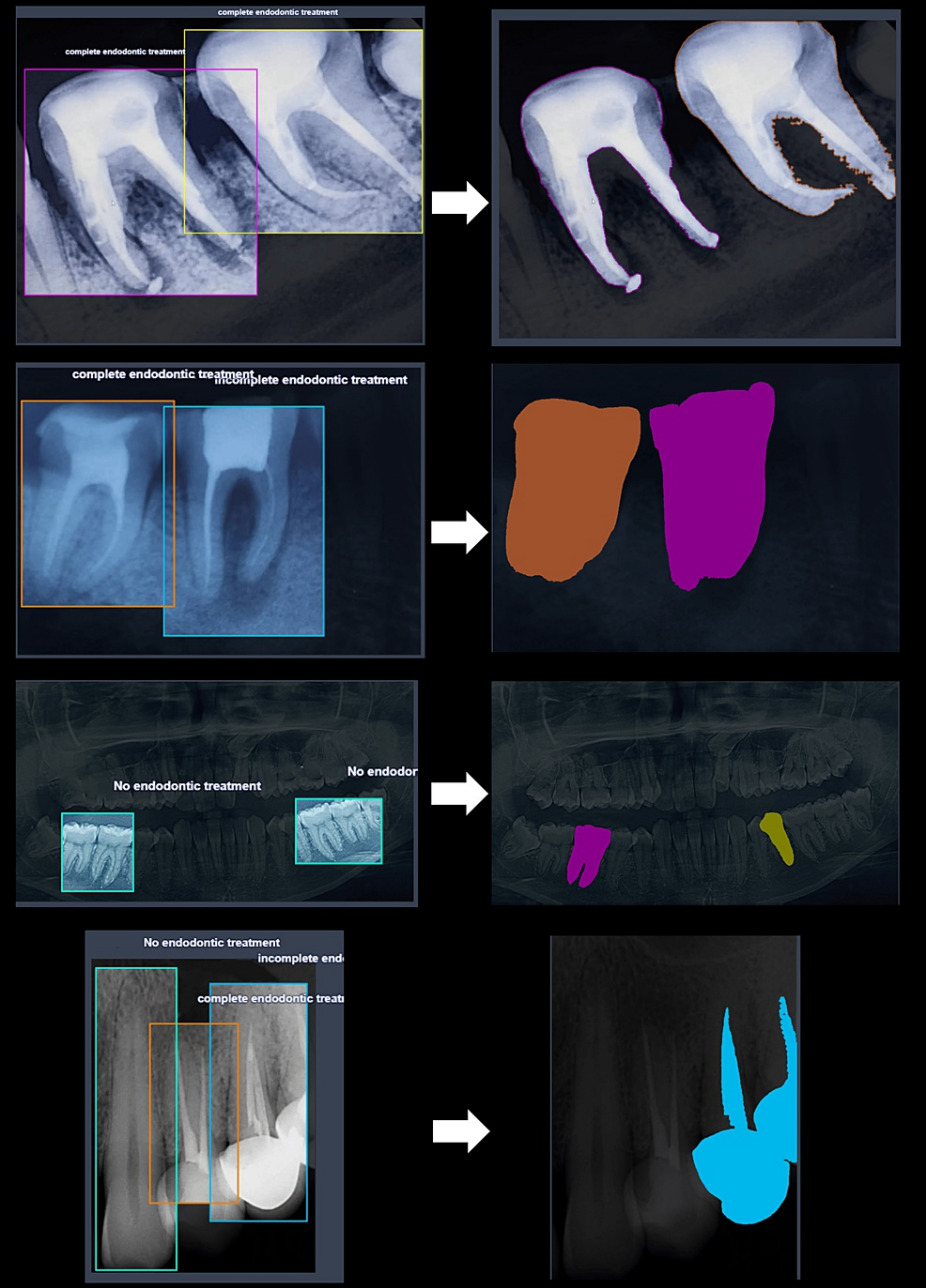
Bounding boxes outlining areas of interest (left) on dental radiographs and the predicted masks for segmentation (right) as derived from CAST outputs CAST: clinical annotation and segmentation tool

Upon the completion of training the YOLOv8 model on a consolidated dataset, a mean average precision (mAP) of 77.4% was successfully attained, accompanied by documented precision, and recall rates of 75.0% and 72.1%, respectively. The subsequent integration of SAM with pre-trained YOLOv8 model weights allowed for the seamless automation of the segmentation process for previously unobserved data. This automated process involved evaluating segmentation capabilities and margins, which were applied to raw, unprocessed data sourced from the radiographic dataset (Figure 3).

**Figure 4:**
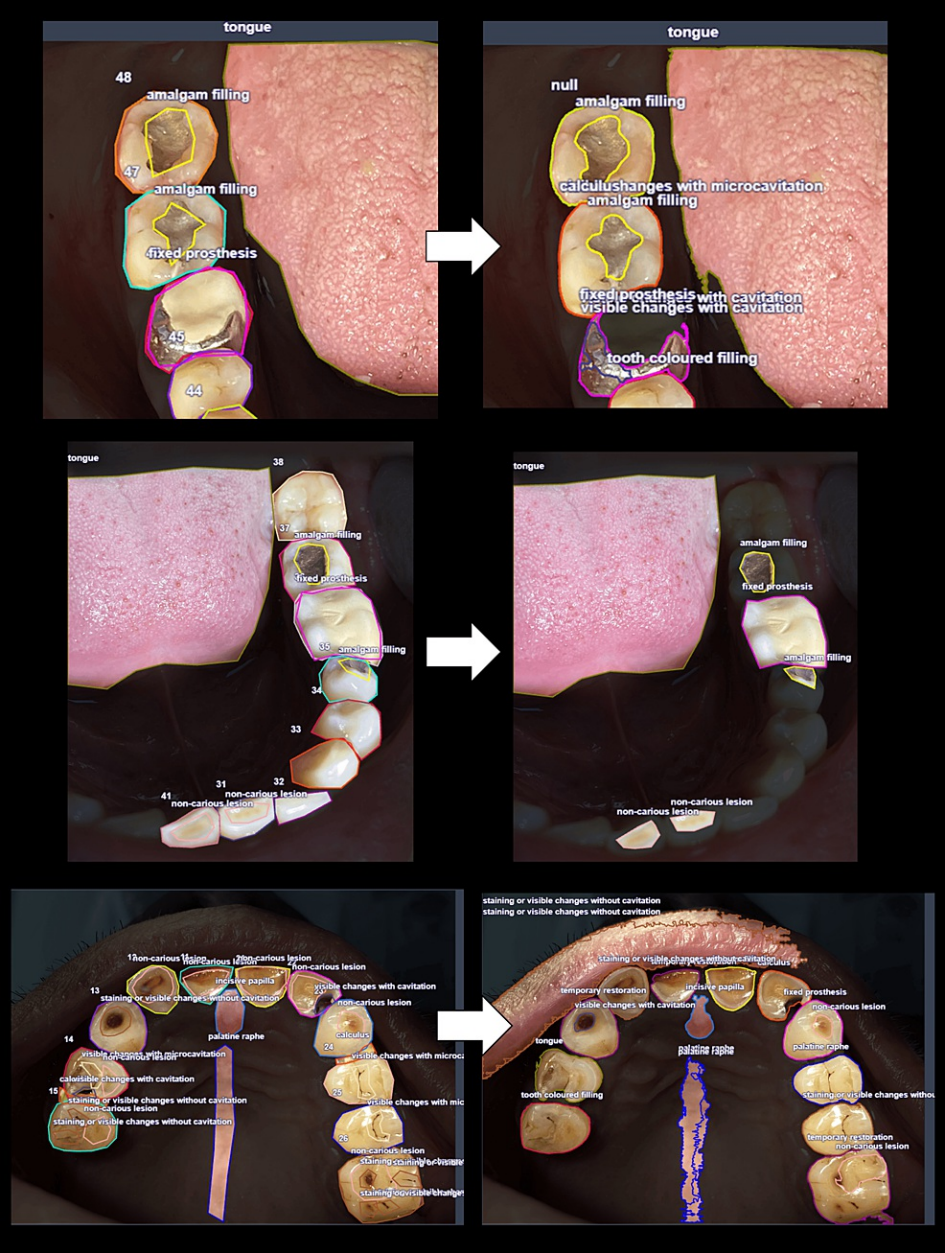
Original polygonal annotations (left) on intraoral photographs and the predicted labels (right) as derived from CAST outputs

Concurrently, a detailed examination was conducted on the replication of labels within intraoral photographs (Figure 4). It is essential to note that this unseen data was not utilised during the training of either of the models.

However, some challenges were encountered. The effectiveness of tooth notations was hindered by the vast variations in occlusal status and the insufficient quantity of data necessary for the proper training of the model. Additionally, challenges arose when multiple teeth of similar classifications were encompassed within a single bounding box in radiographs, leading to incomplete segmentation of individual teeth. This issue was less frequent in intraoral images labelled by masks with polygonal boundaries. Furthermore, the recognition of prostheses, restorations, and non-carious lesions proved to be more reliable compared to the detection of carious lesions that lacked visible cavitation. In some instances, carious changes with cavitation were incorrectly identified as amalgam restorations, presenting a unique challenge in the segmentation process.

## Discussion

The primary objective of this study was to establish a comprehensive auto-annotation and segmentation workflow capable of effectively segmenting clinical features in both intraoral photographs and radiographs. The primary intent of consolidating several advanced AI models into a single stream workflow for multiple datasets was successful. However, it is important to discuss the challenges experienced and possible solutions that may be explored.

As expected, a significant challenge emerged due to the differing methods used to label radiographs and intraoral images. Radiographs were initially labelled using bounding boxes while polygonal boundaries were employed to classify intraoral images. Although both methods have their applications, they also present inherent limitations. For intraoral images and dental radiographs, the use of bounding boxes within crowded regions can lead to inaccurate segmentation [23]. This is owed to the close proximity of each tooth to one another and the divergence of the roots, causing a good portion of one bounding box to overlap with another in order to obtain full coverage of the teeth. SAM demonstrated reasonable proficiency according to the two operators in automatically segmenting tooth morphology based on the bounding boxes but encountered difficulties with radiographs featuring multiple overlapping obturated root canals. However, the proficiency of segmentation could not be quantified owing to the absence of a consistent ground truth. Future comparisons can be made across larger cohorts of clinical operators following further refinement of CAST. The radio-opacity of the obturation often got confused with the radiographic margins of the roots, especially when lamina dura (the compact bone immediately surrounding the tooth root) was not prominently visible. In contrast, polygon-based masking can represent the true shape of an object and tooth structure but does so using a multitude of arbitrary points, which can lead to a general approximation of the object's shape [24]. Unsurprisingly, SAM, which also strives to generate segmented outputs with polygonal boundaries, performed better with intraoral images irrespective of the amount of moisture or salivary pooling on the teeth themselves. When dealing with partially obstructed and ambiguous scenarios, SAM is designed to be ‘ambiguity-aware’. It is able to predict multiple masks for a single prompt to handle ambiguous cases. A mask in the current instance is an area of an image that has different pixel intensities compared to the rest of the image and therefore is visible to object detection models. SAM can therefore generate separate masks for multiple instances, allowing it to handle complex scenes. Of note, SAM has displayed remarkable performance in segmenting aerial images, even when handling intricate details [25]. This proficiency extends to scenarios similar to daytime aerial imaging and the segmentation of water bodies. Further research can explore explainability to gain insight into SAM's segmentation processes when applied to oral images. In some instances, SAM’s abilities to trace the margins of the teeth in intraoral photographs surpassed that of human operators. However, challenges arose when analysing clinical intraoral photographs where shadows were cast, occluding the buccal folds. These regions of low light were eventually mistaken for radiographic datasets and were labelled accordingly. Similar translatory issues were also experienced in radiographs of teeth with obturated canals or unique canal curvatures. As of 2023, this issue is also known to exist in AI applications within other forms of medical imaging, requiring collective efforts for resolution in future research [26].

The study also examined the performance of YOLO as an object detection model in radiographic datasets. Previous investigations revealed that the greatest limitations in this context were noise and class imbalances across the imaging groups, which was also experienced in the current study with the intraoral image dataset [18]. Although having radiographs aligned with clinical photographs was preferred, the authors faced challenges in collecting an adequate number of oral images for the design implementation. Consequently, a noticeable class imbalance existed between oral images and radiographs, with extra radiographs sourced from a previous database unrelated to intraoral images. Furthermore, it is worth noting that intraoral images cannot reliably ascertain the depth of carious lesions [17], which occasionally led SAM to misinterpret corroded amalgam restorations as cavitated lesions. To address these issues, the study proposes manual correction at later stages through reinforcement learning, an established approach to teach AI models through rewards and punishment [27], but which is yet to be documented in caries research and is a potential future study [28].

As the currently proposed methods for dentistry are still in the early stages of research development, several limitations were encountered that can potentially be resolved with further maturation of the field of dental AI. The first challenge to address involved the tracking of canal curvature and obturation thickness in radiographs, a task made even more difficult due to the dataset's previous labelling with bounding boxes by two operators. These bounding boxes often encroached on one another (Figure 1), making reannotation infeasible due to the unavailability of operators and logistical constraints. Furthermore, previous exploratory research on fine-tuning caries diagnostics from digital images was conducted using YOLO versions 4 through 7. However, these knowledge bases could not be seamlessly transferred [29] to YOLOv8, as was initially intended, due to substantial differences in design architecture, effectively limiting the integration of prior findings. Additionally, it is also noteworthy that the current workflow, heavily reliant on the SAM system, proved incompatible with older YOLO versions, necessitating the sole use of YOLOv8 without the opportunity to explore other compatible alternatives. The issues arising from mis-annotations of radiographs and intraoral photographs alike require further investigation for explainability to enhance model transparency [30]. This caused the entire workflow to be heavily dependent on the YOLOv8 and be completely unsupported by the older YOLO versions.

The integration of YOLOv8 and SAM through X-AnyLabeling for human operator-induced reinforced learning involved the conversion of native files containing the labels. txt and json are two common computer file formats that are used in object detection to annotate images. However, they often can not be used interchangeably as they document parameters using specific methods. Integrating the models mentioned in the current study required transitioning from the .txt to .json format, a process with its own set of limitations. .txt's unstructured format complicated data extraction, while .json's verbosity and nested structures could hinder readability and efficiency. The decision to convert the annotated and segmented .txt labels to .json format was primarily driven by discrepancies in data structures and tool requirements. To enhance this process, the potential for establishing a standardised annotation format or developing automated conversion scripts in the future could be explored as possible solutions.

**Figure 5:**
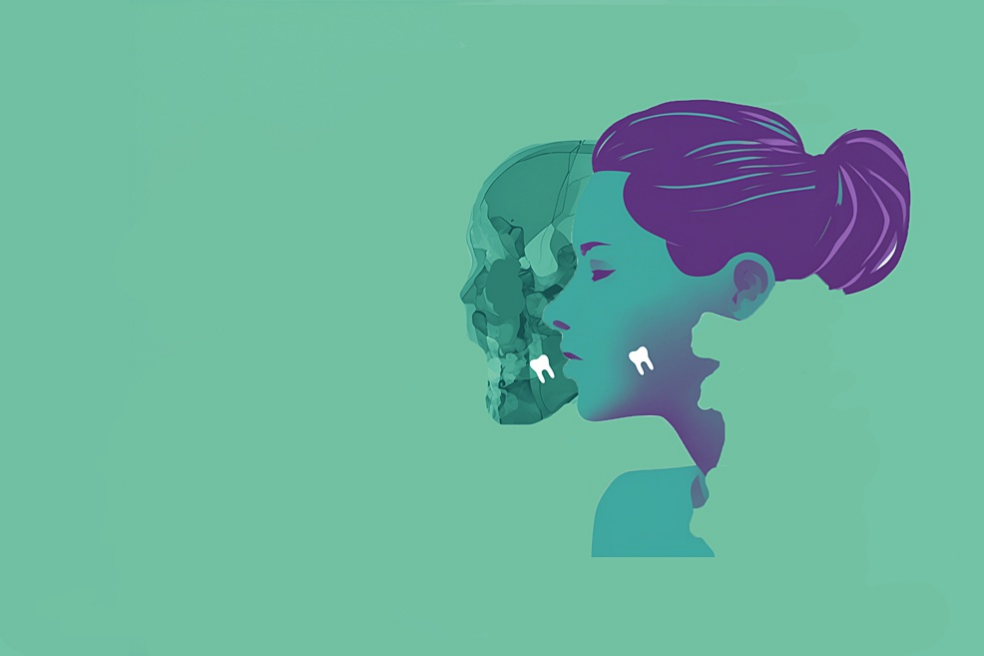
Dental Loop: clinical annotation and segmentation tool (CAST) with proposed reinforcement learning features to characterise and isolate similar features across clinical photographs and oral radiographs

To address the discussed limitations, a potential solution involves the development of an automatic annotation system using reinforcement learning. In this approach, the system defines the state as the current image and utilizes existing annotations generated by the current model. The actions are considered as potential annotations. Reinforcement learning, a machine learning paradigm, allows the model to be rewarded or penalized for its predictions, enabling self-correction. A reward function, assessed by medical experts, can be established to evaluate the quality of annotations. The system initiates with a random policy, a function determining actions for each state, and refines it over time through reinforcement learning. The system learns to strike a balance between exploration, i.e., trying new actions, and exploitation, i.e., using the best-known policy. Figure 5 depicts the graphical illustration of what reinforcing the same data on clinical photography and oral radiographs may represent.

## Conclusions

The development of an AI-based, unsupervised clinical annotation and segmentation tool (CAST) offers the potential to streamline image labelling and segmentation for dental intraoral images and radiographs. This potential is supported by metrics showing precision scores exceeding 70%. However, manually annotated images using bounding boxes face limitations when it comes to segmenting teeth. This is due to the complex anatomic curvature of teeth, their relation to the dental arch, and issues like bounding boxes overlapping when trying to isolate adjacent teeth. Segmenting tooth notations and specific clinical features also remains challenging, even when manual annotations use polygonal boundaries. Furthermore, the tool needs improvement in recognizing shadows in the buccal fold, which are sometimes misinterpreted as radiographs. Explainable AI and reinforcement learning can enhance this recognition.
